# Pharmacogenomics in Orofacial Clefts Care: Insights from Whole-Genome Sequencing of Case-Parents Trios

**DOI:** 10.3390/jpm15100456

**Published:** 2025-09-30

**Authors:** Elvis Poku-Adusei, Gideon Okyere Mensah, Christian Opoku Asamoah, Bruce Tsri, Hafsa Akeeya, Abass Shaibu Danbaki, Solomon Obiri-Yeboah, Tamara D. Busch, Lawrence Sheringham Borquaye, Peter Donkor, Azeez Butali, Lord Jephthah Joojo Gowans

**Affiliations:** 1 Department of Biochemistry and Biotechnology, College of Science, KNUST, Kumasi 00233, Ghana; epokuadusei2@st.knust.edu.gh (E.P.-A.); og.mensah@knust.edu.gh (G.O.M.); coasamoah6@st.knust.edu.gh (C.O.A.); btsri@st.knust.edu.gh (B.T.); hakeeya@st.knust.edu.gh (H.A.); asdanbaki@idl.knust.edu.gh (A.S.D.); 2 Department of Oral and Maxillofacial Sciences, School of Dentistry, KNUST, Kumasi 00233, Ghana; sobiri-yeboah.chs@knust.edu.gh; 3 National Cleft Care Center, Komfo Anokye Teaching Hospital, Kumasi 00233, Ghana; 4 Department of Oral Pathology, Radiology and Medicine, The University of Iowa, Iowa City, IA 52242-1479, USA; tamara-busch@uiowa.edu (T.D.B.);; 5 Department of Chemistry, College of Science, KNUST, Kumasi 00233, Ghana; 6 Department of Surgery, School of Medical Sciences, KNUST, Kumasi 00233, Ghana; petadonkor.chs@knust.edu.gh

**Keywords:** pharmacogenetics, pharmacogenomics, drug metabolism, protein structure, molecular docking, orofacial clefts, African populations

## Abstract

**Background/Objectives**: Orofacial clefts (OFCs) are among the most common birth defects globally, sometimes exacerbated by adverse drug reactions (ADRs) from corticosteroids and antiepileptics. Comprehending the pharmacogenomic and pharmacogenetic elements that lead to ADRs is essential for enhancing precision medicine and clinical outcomes. This study examines rare genetic variants in drug-metabolizing and drug-transporting genes among Ghanaian and Nigerian families with a history of OFCs, intending to assess their pathogenicity and functional implications. **Methods:** We recruited 104 Ghanaian families and 26 Nigerian families, generating whole-genome sequencing (WGS) data from 390 individuals (130 case-parent trios). DNA isolated from saliva and buccal swab samples underwent WGS, and subsequent WGS data were analyzed through extensive bioinformatics analyses. Variants were called and annotated using the GATK workflow. The HOPE in silico modeling tool evaluated the structural impact of genetic variants on encoded proteins, while molecular docking using PyRx examined alterations in ligand binding affinity. **Results**: Our study revealed pathogenic variants in vital genes associated with drug metabolism and transport, specifically *CYP1A2*, *CYP2C18*, *CYP27A1*, *CYP2B6*, *SLC6A2*, and *ABCC3*. Structural modeling research demonstrated substantial size, charge, conformation, and hydrophobicity variations between wildtype and mutant proteins. Variants positioned near conserved regions or within functional domains were anticipated to be deleterious, potentially compromising protein function and ligand interactions. Molecular docking studies verified changes in binding affinities between wildtype and mutant proteins for common ligands. The identified variations were linked to the metabolism of frequently used pharmaceuticals in Africa, such as caffeine, ketoconazole, efavirenz, carbamazepine, and artemether. **Conclusions**: These findings highlight the need for pharmacogenetic screening to inform personalized medicine, diminish ADRs, and enhance the clinical care of OFCs in Sub-Saharan Africa.

## 1. Introduction

Orofacial clefts (OFCs) are the most prevalent craniofacial anomalies globally, characterized by complex genetic and environmental interactions as risk factors. The formation of craniofacial structures involves complex molecular pathways, including bone morphogenetic proteins, sonic hedgehog, and wingless-related integration sites [1]. Numerous genes have been identified in etiologic studies on OFCs. These include *IRF6*, *TBX22*, *MAFB*, *ARHGAP29*, *VAX1*, and *PAX7*, highlighting the genetic complexity associated with these conditions [2]. The classification of OFCs includes various presentations, primarily comprising isolated cleft lip (CL), isolated cleft palate (CP), or their combinations [3]. These abnormalities may manifest unilaterally or bilaterally and can arise as isolated defects (non-syndromic) or as components of wider syndromes. Syndromic types encompass well-documented disorders such as Pierre Robin Sequence, Trisomy 13, Trisomy 18, Apert Syndrome, Stickler Syndrome, and Waardenburg Syndrome [4,5].

Global epidemiological data indicate that OFCs occur in 1 to 2.2 per 1000 births [6]. The incidence exhibits considerable diversity among many people, affected by genetic factors and environmental exposures. Environmental risk factors encompass maternal exposure to teratogens, infections, pharmaceuticals, tobacco use, alcohol intake, radiation, and nutritional deficiencies, especially folate deficiency [3]. Environmental factors interact with genetic predispositions, resulting in differing severities of facial developmental anomalies. Research indicates that newborns with OFCs frequently encounter difficulty in sucking and swallowing, as well as challenges with speech development and social interaction [7]. Dental growth is often impaired, with delays in tooth eruption usually noted in these patients [8].

Despite the surgical treatment of OFCs, patients in Sub-Saharan Africa consume a range of medications, including analgesics, antimalarials, antibacterials, and antiretrovirals, which may culminate in adverse drug reactions (ADRs) due to the generation of reactive metabolites during drug biotransformation. Drug biotransformation occurs in various phases. Phase I reactions primarily involve cytochrome P450 enzymes in the liver. These enzymes enable the integration of polar structures by oxidation, reduction, and hydrolysis of pharmaceutical substances [9]. Phase II activities encompass many transferase enzymes that conjugate modified pharmaceuticals to improve their aqueous solubility and promote excretion [10]. Cytochrome P450 enzymes are pivotal in drug metabolism, accounting for over 75% of enzymatic drug metabolism activities [11]. The CYP superfamily comprises several significant enzymes, including *CYP1A2*, *CYP2C18*, *CYP27A1*, and *CYP2B6*; each characterized by unique substrate specificities and regulatory mechanisms [12]. Moreover, drug transporters such as *SLC6A2* and *ABCC3* are essential for the distribution and removal of drugs.

Pharmacogenomics holds substantial importance in clinical practice, explaining more than 80% of the diversity in pharmacological effectiveness and safety profiles [13]. Genetic polymorphisms can markedly affect pharmacokinetics and pharmacodynamics, resulting in various metabolizer phenotypes: extensive metabolizers (EM), ultra-rapid metabolizers (UM), intermediate metabolizers (IM), and poor metabolizers (PM) [14]. ADRs constitute a significant healthcare issue in Sub-Saharan Africa, especially in Ghana and Nigeria. From 2000 to 2012, Ghana recorded 343 ADRs in children aged 0 to 17 years, leading to 23 fatalities. In comparison, Nigeria reported 473 cases with 21 deaths [15]. These figures underscore the pressing necessity for extensive pharmacogenomic research in African populations, particularly among families impacted by OFCs.

This study aimed to discover and characterize genetic variants in drug-metabolizing and drug-transporting genes in Ghanaian and Nigerian families affected by OFCs. We employed an integrated methodology combining whole-genome sequencing (WGS), protein structure modeling, and molecular docking to assess the structural and functional effects of the variants. This study enhances our understanding of pharmacogenetic diversity in Sub-Saharan African populations and holds significant implications for personalizing pharmacological therapy in patients with OFCs.

## 2. Materials and Methods

### 2.1. Study Design and Population

This cross-sectional study investigated variations in drug metabolism and transport genes across families impacted by OFCs. We recruited 130 case parent trios (104 from Ghana and 26 from Nigeria) from the Komfo Anokye Teaching Hospital (Ghana), Lagos University Teaching Hospital, and Obafemi Awolowo University Teaching Hospital (Nigeria). The sample included 51 cases of cleft lip and 79 cases of cleft lip and palate. All cases were non-syndromic OFCs from simplex pedigrees. The detailed sample descriptions have been published elsewhere [16].

### 2.2. Sample Collection and DNA Extraction

Saliva samples were obtained from parents and probands using Oragene Saliva Kits (DNA GenoTek, Ottawa, ON, Canada). Cheek swab samples were obtained from younger probands employing cotton swabs. DNA extraction followed the Oragene Saliva Protocol, with DNA quantified using the Qubit 4 Fluorometer (http://www.invitrogen.com/site/us/en/home/brands/Product-Brand/Qubit.html (Accessed on 27 June 2023); ThermoFisher Scientific, Grand Island, NY, USA). As a quality control step, the sexes of participants were verified through TaqMan XY genotyping. The detailed protocols have been published elsewhere [16].

### 2.3. Whole-Genome Sequencing and Bioinformatics Analysis

The protocols for WGS, quality control checks, and bioinformatics analysis have been published elsewhere [16], but a summary is given here. DNA samples that met quality control standards were sequenced at the Broad Institute through the Gabriella Miller Kids First Paediatric Research Consortium (https://kidsfirstdrc.org/ , accessed on 10 December 2023). The WGS was conducted at the Broad Institute, with the entire genome sequenced at an average read depth of 30 (30X). Sequence alignment map (SAM) files were obtained after the sequence data were aligned to the Human Genome Assembly GRCh38 (hg38) and converted into binary alignment map (BAM) file format. Alternate alleles (i.e., variants from the reference genome) were called using the GenomeAnalysisToolKit (GATK) pipelines by the Broad Institute (https://software.broadinstitute.org/gatk/best-practices/workflow , accessed on 6 January 2022). Briefly, single-nucleotide variants (SNVs) and insertions or deletions (Indels) were called using the HaplotypeCaller (GATK 4.2.3.0) in GVCF mode for single-sample variant calling and in GenotypeGVCFs for the multiple-sample joint variant calling. Variants were stored in a variant call format (VCF) file, which was used for further analyses. Quality control of VCF files has been published [16]. We filtered for variants with minor allele frequency (MAF < 1%) using databases including the 1000 Genomes Project, Exome Variant Server, dbSNP, and gnomAD [17].

### 2.4. Selection of Genes

We focused on 50 clinically relevant pharmacogenes for Africans (Supplemental Table S1), including *CYP2A6*, *CYP2B6*, *CYP2C8*, *CYP2C9*, *CYP2C19*, *CYP2D6*, *CYP3A4*, *CYP3A5*, *NAT*, *UGT*, *ABC*, and *SLC* families. These genes were selected based on their classification as Very Important Pharmacogenes by PharmGKB [18] and their documented genetic variability in African populations [19].

### 2.5. Variant Classification and Structural Analysis

Variants were classified according to American College of Medical Genetics and Genomics (ACMG) recommendations [20], using ClinVar and eleven other variant effect predictive tools embedded in dbNSFP [21]. Pathogenicity of missense variants was assessed using twelve tools, including SIFT, PolyPhen-2, Mutation Taster, Mutation Assessor, MetaRNN, REVEL, MutPred, BayesDel_addAF, ClinPred, CADD, ClinVar, and AlphaMissense. Missense variants were considered pathogenic if at least six of these tools, in addition to ClinVar, predicted such (Supplementary Tables S2 and S3). Loss-of-function (LOF) variants were evaluated based on CADD and REVEL, whereas SpliceAI (accessed on 22 Feburary 2024) was used to evaluate splice site variants. Selected variants were subjected to further investigation using segregation analysis and functional assessment.

The HOPE [22] analysis toolkit was utilized for in silico modeling of protein tertiary structure to evaluate how gene variants influence the structure and function of affected proteins. The HOPE methodology begins with inputting the protein sequence in FASTA format or as an accession code through an easy-to-use web interface. A BLAST (v2.15.0) analysis is then conducted to identify similar sequences in the UniProt [23] database and search for 3D structures or templates in the Protein Data Bank (PDB) for homology modeling. If no direct structure is available, Yasara (v25.1.13) software [24] is employed for homology modeling to generate a 3D model automatically. The analysis proceeds with data collection, where structural characteristics such as residue accessibility, secondary structure, and ligand interactions are examined using WHAT IF web services [25]. Functional information, including active sites, domains, and motifs, is retrieved from UniProt annotations. Additionally, Reprof [26] predictions are used to provide additional insights into secondary structure and solvent accessibility if necessary. The data gathered is then integrated through a decision tree, which prioritizes the most reliable sources, such as real protein structures, UniProt annotations, and Reprof predictions. This synthesized information is used to generate a report that assesses the impact of the variant on structural contacts, functional regions, post-translational modifications, variations, and amino acid properties. The final output is presented in an accessible and user-friendly format [22].

Molecular docking simulations were performed using protein structures derived from AlphaFold [27] and ligands sourced from PubChem [28]. Protein preparation was performed using Discovery Studio [29] to ensure structural optimization and minimization. Mutant protein variants were generated using Chimera [30], utilizing its mutagenesis tools to simulate single-nucleotide polymorphisms and assess their structural effects. Ligand libraries were curated and refined in Spartan 14 [31] using its quantum chemical calculation features to optimize molecular geometries and electronic characteristics. Docking simulations were performed globally with PyRx [32], enabling the screening and ranking of ligands based on their binding affinities to both the wildtype and mutant proteins. The top protein–ligand complexes with the best docking poses were visualized and analyzed using Discovery Studio [29] and Chimera [30]. Key structural interactions were identified to offer insights into binding modes and potential functional consequences of the variant.

## 3. Results

### 3.1. Identification and Characterization of Pathogenic Variants in Drug-Metabolizing and Drug-Transporting Genes

Our thorough analyses of WGS data from families with OFCs identified several pathogenic variations in genes essential for drug metabolism and transport. Fifty-seven (57) variants in fifty (50) drug-metabolizing and transporting genes were observed (Table 1, Supplemental Table S6). The pathogenicity of the variant was meticulously evaluated utilizing twelve prediction tools: SIFT, PolyPhen-2, Mutation Taster, Mutation Assessor, MetaRNN, REVEL, MutPred, BayesDel_addAF, ClinPred, CADD, ClinVar, and AlphaMissense. We established stringent standards whereby missense variants were designated pathogenic only when they were predicted by over five tools consistently (Supplemental Table S2). Supplemental Table S3 outlines the syndromes, metabolic pathways, and commonly used drugs or substrates metabolised by the genes with pathogenic variants. Supplemental Table S4 outlines the minor allele frequencies (MAFs) of the variants in the current study and other populations of African ancestry.

### 3.2. Structural and Functional Impact Analysis

#### 3.2.1. *CYP1A2* Variants

In the *CYP1A2* gene, we detected two pathogenic variants (Table 2): rs45565238 (c.217G>A, p.Gly73Arg) and a novel variant (c.269G>C, p.Arg90Pro). This gene is linked to many diseases, including Porphyria Cutanea Tarda and Hepatocellular Adenoma, exhibiting autosomal dominant inheritance (Table S5). The comprehensive HOPE analysis (Table 2) indicated that the p.Gly73Arg mutation in *CYP1A2* considerably affected protein flexibility at a highly conserved site. The mutation introduced a larger, positively charged residue, compromising the protein’s structural stability. The p.Arg90Pro variant modified hydrogen bonding patterns and hydrophobicity, presumably influencing substrate identification and binding.

#### 3.2.2. *CYP2C18* Variants

Two variants in *CYP2C18* were identified: rs59636573 (c.988G>T, p.Val330Leu) and rs60181876 (c.896C>T, p.Thr299Ile), Table 1. This gene is associated with Danubian Endemic Familial Nephropathy and Coumarin Resistance, exhibiting an autosomal dominant inheritance pattern (Table S5). The p.Val330Leu variant caused alterations in secondary structure preferences. However, the projected protein damage was minor. The p.Thr299Ile variant, situated near a highly conserved site, introduced a bigger, more hydrophobic residue, which may influence protein–substrate interactions (Table 2).

#### 3.2.3. *CYP27A1* Variants

In *CYP27A1*, we detected rs145722193 (c.1102G>T, p.Val368Leu) and rs151117761 (c.1564G>A, p.Val522Met), Table 1. This gene is linked to metabolic illnesses, including Xanthomatosis and Lipid Storage Disease, exhibiting autosomal recessive inheritance (Table S5). The p.Val368Leu and p.Val522Met variants in *CYP27A1* influenced β-strand preferences and introduced larger residues, possibly leading to structural perturbations.

#### 3.2.4. *CYP2B6* Variants

The *CYP2B6* gene had two variants: rs764288403 (c.1301G>A, p.Arg434Gln) and rs372295360 (c.293G>A, p.Arg98Gln), Table 1. This gene has been associated with Neonatal Abstinence Syndrome, which exhibits X-linked dominant inheritance (Table S5). The *CYP2B6* variants (p.Arg434Gln and p.Arg98Gln) had substantial impacts on ligand binding and protein stability, with the Arg434Gln variant loss of positive charge disrupting protein electrostatic interaction with negatively charged residues or co-factors (Table 2).

### 3.3. Molecular Docking Analysis and Drug-Binding Implications

#### 3.3.1. *CYP1A2* Drug Interactions

**Figure 1:**
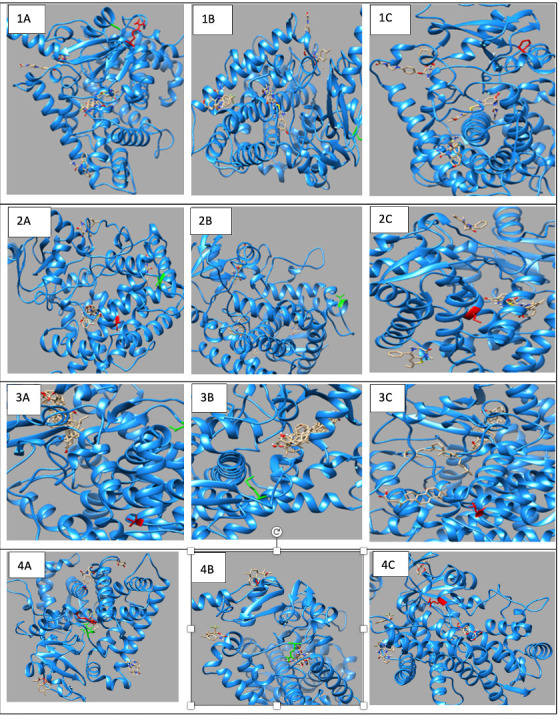
Molecular Docking Analysis and Drug-Binding Implications on Drug Metabolizers: (**1**): A 3D interaction of ligands to wildtype and variants of *CYP1A2.* (**A**) A 3D interaction of wildtype *CYP1A2* with ligands (Green: wildtype Glycine; Red: wildtype Arginine). (**B**) A 3D interaction of Gly73Arg variant *CYP1A2* with ligands (Green: mutant Arginine). (**C**) A 3D interaction of Arg90Pro variant *CYP1A2* with ligands (Red: mutant Proline). (**2**): A 3D interaction of ligands to wildtype and variants of *CYP2C18*. (**A**) A 3D interaction of wildtype *CYP2C18* with ligands (Green: wildtype Valine; Red: wildtype Threonine). (**B**) A 3D interaction of the Val330Leu variant of *CYP218* with ligands (Green: mutant Leucine). (**C**) A 3D interaction of the Thr299Ile variant of *CYP2C18* with ligands (Red: mutant Isoleucine). (**3**): A 3D interaction of ligands to wildtype and variants of *CYP27A1*. (**A**) A 3D interaction of wildtype *CYP27A1* with ligands (Green: wildtype Valine; Red: wildtype Valine). (**B**) A 3D interaction of the Val368Leu variant of *CYP27A1* with ligands (Green: mutant Leucine). (**C**) A 3D interaction of the Val522Met variant of *CYP27A1* with ligands (Red: mutant Methionine). (**4**): A 3D interaction of ligands to wildtype and variants of *CYP2B6*. (**A**) A 3D interaction of wildtype *CYP2B6* with ligands (Green: wildtype Arginine; Red: wildtype Arginine). (**B**) A 3D interaction of the Arg434Gln variant of *CYP2B6* with ligands (Green: mutant Glutamine). (**C**) A 3D interaction of the Arg98Gln variant of *CYP2B6* with ligands (Red: mutant Glutamine).

Molecular docking investigations demonstrated substantial alterations in substrate binding (Figure 1, Table S7). The wildtype *CYP1A2* exhibited optimal binding with omeprazole (−8.5), but the Gly73Arg mutant had enhanced affinity (−9.0). Both variants exhibited decreased affinity for caffeine (wildtype: −7.2; Gly73Arg: −6.9; Arg90Pro: −6.0) and fluvoxamine (wildtype: −7.7; Gly73Arg: −6.2 and Arg90Pro: −6.0).

#### 3.3.2. *CYP2C18* Substrate Binding

The Val330Leu variant showed altered binding patterns with key substrates (Figure 1, Table S8). Notable changes included reduced affinity for omeprazole (wildtype: −7.7; Val330Leu: −6.9) and tolbutamide (wildtype: −6.1; Val330Leu: −5.9). The Thr299Ile variant maintained similar binding affinities for most substrates but showed enhanced binding to tolbutamide (−6.9).

#### 3.3.3. *CYP27A1* Metabolic Effects

Docking studies demonstrated complex impacts on substrate binding (Figure 1, Table S9). The Val368Leu variant exhibited diminished affinity for chenodeoxycholic acid (−7.7 compared to wildtype −8.8) but enhanced affinity for ergocalciferol (−9.9 compared to wildtype −9.6). The Val522Met variant exhibited increased binding affinity to cholecalciferol (−9.4 compared to wildtype −9.1).

#### 3.3.4. *CYP2B6* Metabolic Effects

Docking studies demonstrated distinct impacts on substrate binding (Figure 1, Table S10). *CYP2B6* showed significant changes in binding affinities, particularly with efavirenz, where affinity decreased from −8.3 (wildtype) to −7.3 (Arg434Gln) and −7.2 (Arg98Gln), while nevirapine showed improved binding in the Arg98Gln variant (−8.4) compared to wildtype (−6.6).

### 3.4. Drug Transport Gene Variants

**Figure 2:**
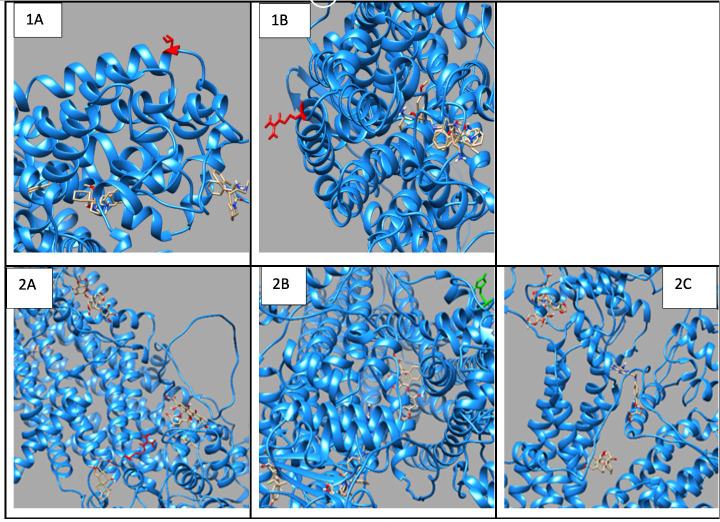
Molecular Docking Analysis and Drug-Binding Implications on Drug Transporters. (**1**): A 3D interaction of ligands to the wildtype and variant of *SLC6A2*. (**A**) A 3D interaction of wildtype *SLC6A2* with ligands (Red: wildtype Threonine). (**B**) A 3D interaction of the p.Thr283Arg variant of *SLC6A2* with ligands (Red: mutant Arginine). (**2**): A 3D interaction of ligands to wildtype and variants of *ABCC3*. (**A**) A 3D interaction of wildtype *ABCC3* with ligands (Red: wildtype Arginine). (**B**) A 3D interaction of Arg1297His variant of *ABCC3* with ligands (Green: mutant Histidine). (**C**) A 3D interaction of the Arg1166Cys variant of *ABCC3* with ligands (Red: mutant Cysteine).

The p.Thr283Arg variant of *SLC6A2* exhibited notably constant binding affinities for inhibitors of neurotransmitter transport, indicating preserved functionality despite the mutation (Figure 2; Table S11). Conversely, *ABCC3* variants (Figure 2, Table S12) exhibited substrate-specific effects, with the p.Arg1166Cys variant revealing significantly altered binding patterns for different drugs, particularly enhanced affinity for dexamethasone (−8.6 compared to wildtype −8.0) and diminished affinity for methotrexate (−8.4 compared to wildtype −8.9). The findings indicate that genetic differences in drug-metabolizing enzymes and transporters might substantially influence drug–protein interactions, potentially impacting therapeutic efficacy and metabolism in persons with OFCs. The noted alterations in binding affinities and protein conformation offer significant insights for personalised medicine strategies for treating patients with these genetic variants.

## 4. Discussion

The identification and characterization of genetic variants in genes encoding drug-metabolizing enzymes and drug-transporting proteins within Ghanaian and Nigerian families with OFCs provides essential insights into the molecular foundations of pharmacogenetic diversity in Sub-Saharan African populations. Our findings indicate diverse genetic variations that may substantially influence medication metabolism and transport, carrying considerable implications for medical treatments in this population. The *CYP1A2* variants observed in our study can potentially modify drug metabolism patterns. The p.Gly73Arg variant, located adjacent to a highly conserved area, induces structural alterations that our modelling indicates may diminish the protein’s catalytic effectiveness. This finding supports earlier research by others [33], which revealed that mutations closer to conserved areas of *CYP1A2* can substantially influence its metabolic capacity. The p.Arg90Pro variant is an undocumented alteration for which our structural study indicates may significantly affect protein function by altering hydrogen bonding networks and hydrophobicity patterns.

Our findings regarding *CYP2B6* variants are particularly relevant due to the enzyme’s function in the metabolism of antiretroviral medicines often used in African populations. The p.Arg434Gln and p.Arg98Gln variants may modify the metabolism of medicines like efavirenz and nevirapine, which are frequently employed in treatments of HIV. These findings expand upon the research conducted by others [34], who highlighted the significance of *CYP2B6* polymorphisms in the outcomes of antiretroviral therapy. Our molecular docking investigations indicate that these variants may influence drug-binding affinity, possibly requiring dose modifications in afflicted people.

The identified variants in *CYP27A1* are significant due to their possible effects on vitamin D metabolism and cholesterol regulation. The p.Val368Leu and p.Val522Met variants exhibit modified binding affinities for critical substrates, indicating possible implications for endogenous metabolism and pharmacokinetics. These findings strengthen the research conducted by others [35], which identified the function of CYP27A1 in cholesterol metabolism and vitamin D activation. Variants observed in transport proteins such as *SLC6A2* and *ABCC3* indicate possible effects on medication distribution and cellular efflux. The p.Thr283Arg variant in *SLC6A2* may influence the transport of many medicinal drugs, whereas *ABCC3* mutations may alter the efflux of conjugated drug metabolites. These findings align with the studies conducted elsewhere [36], which illustrated the significance of these transporters in drug disposal and therapeutic results.

Our structural analyses offer mechanistic insights into the possible impact of these variants on protein function. The identified alterations in amino acid characteristics, protein structure, and ligand interaction indicate various methods by which these variants may affect drug metabolism and transport. These findings expand sequence variation to offer a comprehensive understanding of potential functional implications, facilitating the advancement of more targeted therapeutic strategies. The implications of these findings for therapeutic treatment in Sub-Saharan Africa are substantial. The prevalence of potentially functional variants in essential drug-metabolizing enzymes and drug-transporting proteins indicates the necessity for pharmacogenetic screening prior to starting particular pharmacological therapy, especially for drugs with narrow therapeutic parameters. This method may mitigate the occurrence of ADRs, which is recognised as a major contributor to morbidity in African populations [37].

There is currently insufficient clinical and functional genomics evidence to link specific variants observed in the pharmacogenes in this study to altered teratogenic risk. Nonetheless, the molecular docking analyses provide valuable insights into substrate-specific effects of these variants. The observed changes in binding affinities for common therapeutic agents suggest that certain drugs may require dosage adjustments in individuals carrying these variants. For instance, the altered binding patterns observed with antiepileptic drugs like carbamazepine in *CYP1A2* variants align with clinical observations elsewhere [38], which reported variable drug responses in populations with similar genetic variations. These findings emphasize the importance of considering genetic variation in drug metabolism when designing treatment protocols for patients such as pregnant mothers, for whom ADRs emanating from such variants, as observed in the current study, can predispose their foetuses to OFCs [39]. Of particular concern is the impact of these variants on commonly prescribed medications in African populations. The reduced binding affinity observed for efavirenz in *CYP2B6* variants supports findings by others [40], who reported altered drug metabolism in patients with *CYP2B6* polymorphisms. Similarly, the modified interactions between *ABCC3* variants and various substrates, including acetaminophen and methotrexate, suggest potential implications for the safety and efficacy of these widely used medications. These observations are particularly relevant given the findings of a study [15] that documented significant numbers of ADRs in Ghanaian and Nigerian paediatric populations.

Our findings significantly explain how genetic variants, such as those in *CYP1A2*, affect drug metabolism, highlighting the complexity of genotype–phenotype relationships. Reduced binding affinities for some ligands suggest a loss-of-function effect, whereas increased affinity for others points to potential gain-of-function scenarios. Such duality emphasizes the importance of comprehensive functional assays to fully characterize the molecular pathological basis of variants of pharmagenomics interest, such as those in *CYP1A2*.

Identifying novel variants and previously unreported functional impacts highlights the genetic diversity within African populations and underscores the importance of population-specific pharmacogenetic studies. A study [41] noted that understanding population-specific genetic variation is crucial for developing effective and efficient precision medicine approaches. Our findings contribute to this knowledge base and suggest that current drug dosing guidelines, often developed based on studies in non-African populations, may need revision for optimal application in Sub-Saharan African contexts. The structural changes observed in these proteins also broadly affect our understanding of protein–drug interactions. The detailed characterization of how specific amino acid substitutions affect protein structure and function provides valuable insights for predicting the impacts of other variants in these and related proteins. This knowledge could be particularly valuable for future drug development efforts, as suggested by a study [12], which emphasized the importance of structural understanding in drug design and optimization.

The study had some limitations that highlight areas for future research and improvement. Firstly, the relatively small cohort of 130 families may limit the generalizability of the findings among Africans, necessitating a larger sample size that is more representative of all Africans in future studies in order to better estimate the prevalence of genetic variants linked to ADRs. We did not undertake any longitudinal study to assess whether any of the individuals with the reported likely pathogenic variants had ADRs. For mothers harboring pathogenic variants in the reported pharmacogenes, the possible teratogenic role of ADRs in OFC aetiology in the affected child could not be assessed, since it will take a cohort study to gather such datasets. In silico modelling techniques, such as HOPE analysis and PyRx docking simulations, were instrumental in predicting structural and functional impacts of genetic variants. However, these tools have inherent limitations. These include reliance on theoretical predictions, exclusion of post-translational modifications, and lack of consideration for complex physiological environments. Additionally, the study concentrated on specific drug-metabolizing and drug-transporting genes, potentially overlooking other relevant genetic factors. Its focus on drugs commonly used in Ghana and Nigeria limits applicability to other healthcare contexts.

## 5. Conclusions

Our study provides a comprehensive molecular characterization of genetic variants affecting drug metabolism and transport in Ghanaian and Nigerian families with OFCs. The identified structural and functional impacts of these variants have significant implications for drug therapy in this population. These findings support the need for pharmacogenetic screening in clinical practice and suggest opportunities for optimizing drug therapy through genotype-guided dosing approaches. Implementation of these insights could contribute to reduced ADRs and improved therapeutic outcomes in Sub-Saharan African populations.

## Figures and Tables

**Table 1:** Pathogenic variants observed in genes involved in drug metabolism and transport.

GENE	Genomic Coordinate	Genotype of Proband	HGVSc	HGVSp	Variant in Father? (Genotype)	Variant in Mother? (Genotype)	Number of Tools That Predicted Pathogenicity	Prevalence
								Families (Individuals)
*CYP1A2*	15:74749955 (rs45565238)	G/A	NM_000761.5:c.217G>A	p.Gly73Arg	Yes (G/A)	None	8	2 (4)
	15:74750007 (Novel)	G/C	NM_000761.5:c.269G>C	p.Arg90Pro	Yes (G/C)	None	6	1 (2)
*CYP2C18*	10:94724372 (rs59636573)	G/T	NM_000772.3:c.988G>T	p.Val330Leu	None	Yes (G/T)	6	2 (4)
	10:94720472 (rs60181876)	C/T	NM_000772.3:c.896C>T	p.Thr299Ile	Yes (C/T)	None	6	2 (4)
*CYP27A1*	2:218814105 (rs145722193)	G/T	NM_000784.4:c.1102G>T	p.Val368Leu	None	Yes (G/T)	6	1 (2)
	2:218814998 (rs151117761)	G/A	NM_000784.4:c.1564G>A	p.Val522Met	Yes (G/A)	None	6	2 (4)
*CYP2B6*	19:41016652 (rs764288403)	G/A	NM_000767.5:c.1301G>A	p.Arg434Gln	None	Yes (G/A)	8	1 (2)
	19:41004122 (rs372295360)	G/A	NM_000767.5:c.293G>A	p.Arg98Gln	Yes (G/A)	None	9	1 (2)
*SLC6A2*	16:55691982 (rs45564432)	C/G	NM_001043.3:c.848C>G	p.Thr283Arg	Yes (C/G)	None	6	2 (4)
*ABCC3*	17:50683692 (rs11568591)	G/A	NM_003786.4:c.3890G>A	p.Arg1297His	Yes (G/A)	None	6	3 (6)
	17:50677861 (rs34620384)	C/T	NM_003786.4:c.3496C>T	p.Arg1166Cys	None	Yes (C/T)	6	2 (4)

**Table 2:** Structural and Functional Impact Analysis.

Gene	Variants	2D Depiction of Amino Acid Change	3D Depiction of Amino Acid Change
*CYP1A2*	15:74749955 (rs45565238)	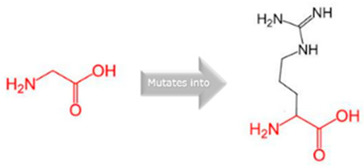 Gly into an Arg at position 73	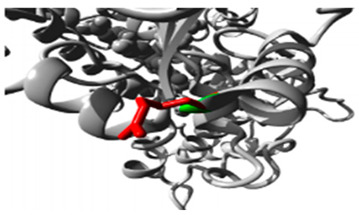 Green: Wildtype (Glycine) Red: Mutant (Arginine)
	15:74750007 (Novel)	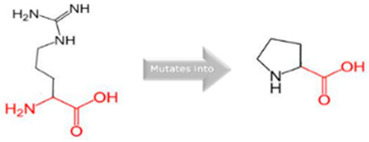 Arg into a Pro at position 90	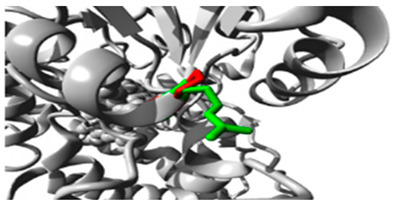 Green: Wildtype (Arginine) Red: Mutant (Proline)
*CYP2C18*	10:94724372 (rs59636573)	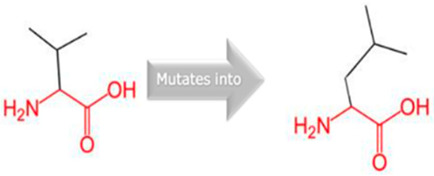 Val into a Leu at position 330	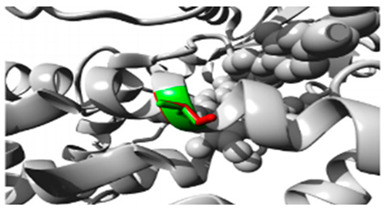 Green: Wildtype (Valine) Red: Mutant (Leucine)
	10:94720472 (rs60181876)	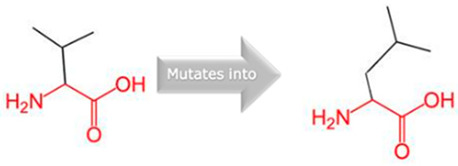 Thre into an Iso at position 299	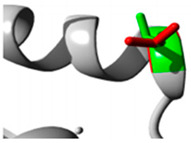 Green: Wildtype (Threonine) Red: Mutant (Isoleucine)
*CYP27A1*	2:218814105 (rs145722193)	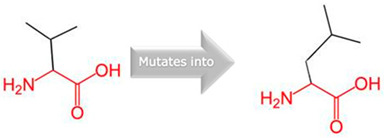 Val into a Leu at position 368	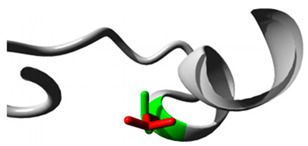 Green: Wildtype (Valine) Red: Mutant (Leucine)
	2:218814998 (rs151117761)	NA	NA
*CYP2B6*	19:41016652 (rs764288403)	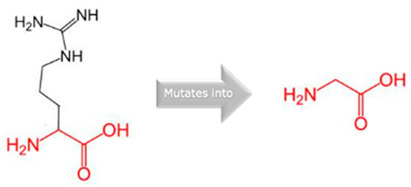 Arg into a Gln at position 434	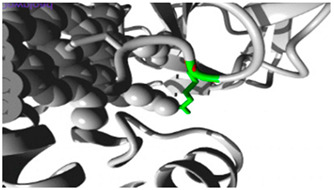 Green: Wildtype (Arginine) Red: Mutant (Glutamine)
	19:41004122 (rs372295360)	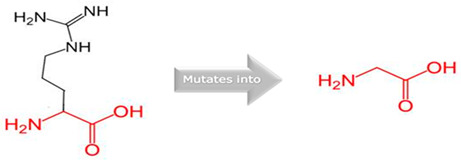 Arg into a Gln at position 98	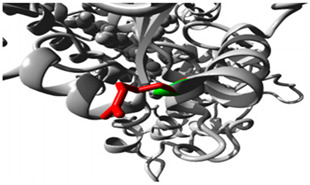 Green: Wildtype (Arginine) Red: Mutant (Glutamine)

## Data Availability

The original data presented in the study are openly available in the Gabriella Miller Kids First Pediatric Research program at https://kidsfirstdrc.org (Accessed on 10 December 2023).

## Supplementary Materials

The following supporting information can be downloaded at: https://www.mdpi.com/article/10.3390/jpm15100456/s1, Table S1: Prioritized pharmacogenes for the study; Table S2: Variant annotation to test deleteriousness of variants using various bioinformatics tools; Table S3: Range and Predictive threshold of tools for testing variant deleteriousness; Table S4: Minor allele frequency variants Table S5: Syndromes, metabolic pathways and common substrates associated with genes with pathogenic variants; Table S6: Other Variants observed in the 50 genes of interest; Table S7: Binding affinity of ligands to *CYP1A2* wildtype and variants; Table S8: Binding affinity of *CYP2C18* wildtype and variants to ligands; Table S9: Binding affinity of *CYP27A1* wildtype and variants to ligands; Table S10: Binding affinity of *CYP2B6* wildtype and variants to ligands; Table S11: Binding affinity of *SLC6A2* wildtype and variant to ligands; Table S12: Binding affinity of *ABCC3* wildtype and variants to ligands.

## Abbreviations

The following abbreviations are used in this manuscript:

ADR	Adverse drug reaction
ACMG	American College of Medical Genetics and Genomics
CL	Cleft lip
CP	Cleft palate
GoF	Gain-of-function
LoF	Loss-of-function
OFC	Orofacial clefts
WGS	Whole-genome sequencing
