# Supplementary material for: Pharmacogenomics in Orofacial Clefts Care: Insights from Whole-Genome Sequencing of Case-Parents Trios
Source: J Pers Med. 2025 Sep 30;15(10):456. doi: 10.3390/jpm15100456 (PMC12565010; doi:10.3390/jpm15100456)
Supplement: Supplementary file 1 [file jpm-15-00456-s001.zip › jpm-3794577-supplementary.pdf]

Supplementary Data

Supplementary tables

Table S1: **Prioritized pharmacogenes for the study.**

**Drug Metabolizing Genes**		**Drug Transporting Genes**	
CYP1A1	cytochrome P450 family 1 subfamily A member 1	***NAT2***	N-acetyltransferase 2
CYP1A2	cytochrome P450 family 1 subfamily A member 2	***FAAH***	fatty acid amide hydrolase
CYP1B1	cytochrome P450 family 1 subfamily B member 1	***UGT1A1***	UDP glucuronosyltransferase family 1 member A1
CYP2A6	cytochrome P450 family 2 subfamily A member 6	***UGT1A3***	UDP glucuronosyltransferase family 1 member A3
CYP2B6	cytochrome P450 family 2 subfamily B member 6	***UGT1A4***	UDP glucuronosyltransferase family 1 member A4
CYP2C8	cytochrome P450 family 2 subfamily C member 8	***UGT1A6***	UDP glucuronosyltransferase family 1 member A6
CYP2C9	cytochrome P450 family 2 subfamily C member 9	***UGT1A7***	UDP glucuronosyltransferase family 1 member A7
CYP2C18	cytochrome P450 family 2 subfamily C member 18	***UGT1A9***	UDP glucuronosyltransferase family 1 member A9
CYP2C19	cytochrome P450 family 2 subfamily C member 19	***UGT1A10***	UDP glucuronosyltransferase family 1 member A10
CYP2D6	cytochrome P450 family 2 subfamily D member 6	***UGT2B15***	UDP glucuronosyltransferase family 2 member B15
CYP2E1	cytochrome P450 family 2 subfamily E member 1	***UGT2B7***	UDP glucuronosyltransferase family 2 member B7
CYP2J2	cytochrome P450 family 2 subfamily J member 2	***ABCA1***	ATP binding cassette subfamily A member 1
CYP3A4	cytochrome P450 family 3 subfamily A member 4	***ABCB1***	ATP binding cassette subfamily B member 1
CYP4B1	cytochrome P450 family 4 subfamily B member 1	***ABCB11***	ATP binding cassette subfamily B member 11
CYP4F2	cytochrome P450 family 4 subfamily F member 2	***ABCC1***	ATP binding cassette subfamily C member 1
CYP7A1	cytochrome P450 family 7 subfamily A member 1	***ABCC2***	ATP binding cassette subfamily C member 2
CYP11B2	cytochrome P450 family 11 subfamily B member 2	***ABCC3***	ATP binding cassette subfamily C member 3
CYP19A1	cytochrome P450 family 19 subfamily A member 1	***ABCC6***	ATP binding cassette subfamily C member 6
CYP27A1	cytochrome P450 family 27 subfamily A member 1	***ABCC8***	ATP binding cassette subfamily C member 8
ADH1A	alcohol dehydrogenase 1A (class I), alpha polypeptide	***SLC6A2***	solute carrier family 6 member 2
ADH1B	alcohol dehydrogenase 1B (class I), beta polypeptide	***SLC6A3***	solute carrier family 6 member 3
ADH1C	alcohol dehydrogenase 1B (class I), beta polypeptide	***SLC6A4***	solute carrier family 6 member 4
ALDH1A1	aldehyde dehydrogenase 1 family member A1	***SLC47A1***	solute carrier family 47 member 1
ALDH2	aldehyde dehydrogenase 2 family member	***SLC29A4***	solute carrier family 29 member 4
CBR1	carbonyl reductase 1	***CBR3***	Carbonyl reductase 3

Table S2: Variant annotation to test deleteriousness of variants using various bioinformatics tools (SIFT, PolyPhen-2, Mutation Taster, Mutation Asssessor, MetaRNN, REVEL, MutPred, BayesDel_addAF, ClinPred, CADD, Clinvar and AlphaMissense).

Genename (rs_dbSNP)	chromosome position	Ensembltranscriptid (Canonical)	HGVSc VEP	HGVSp VEP	SIFT_ score	Polyphen2 score	Mutation Taster score	Mutation Assessors core	MetaRNN score	REVEL score	MutPred score	BayesDel _addAF score	ClinPred score	CADD Score	Alpha Missense score
CYP2C18 (rs60181876)	10/ 94720472	ENST00000285979 (Yes)	c.896C>T	p.Thr299Ile	0.03	0.993	0.34275	3.075	0.018898	0.476	NA	-0.3697	0.126408	21.6	0.5793
CYP2C18 (rs59636573)	10/94724372	ENST00000285979 (Yes)	c.988G>T	p.Val330Leu	0.001	0.998	0.37585	4.045	0.012383	0.572	0.82	-0.28957	0.097447	19.15	0.47
CYP1A2 (rs45565238)	15/74749955	ENST00000343932 (Yes)	c.217G>A	p.Gly73Arg	0	NA	0.81001	4.485	0.827834	0.95	0.921	0.130559	0.326851	26.8	0.5488
CYP1A2 (Novel)	15/74750007	ENST00000343932 (Yes)	c.269G>C	p.Arg90Pro	0.001	NA	0.36238	0.55	0.315314	0.422	0.656	0.0062	0.892129	28.7	0.8073
SLC6A2 (rs45564432)	16/55691982	ENST00000379906 (NA)	c.848C>G	p.Thr283Arg	0.001	0.994	0.81001	3.62	0.017958	0.71	NA	-0.06496	0.042676	26.2	0.2242
ABCC3 (rs34620384)	17/50677861	ENST00000285238 (Yes)	c.3496C>T	p.Arg1166Cys	0.029	1	0.81001	2.345	0.164768	0.627	NA	0.02299	0.101821	28.2	0.1488
ABCC3 (rs11568591)	17/50683692	ENST00000285238 (Yes)	c.3890G>A	p.Arg1297His	0.001	1	0.81001	2.165	0.011793	0.653	NA	-0.23569	0.028615	26.7	0.1365
CYP2B6 (rs372295360)	19/41004122	ENST00000324071 (NA)	c.293G>A	p.Arg98Gln	0.001	0.999	0.31922	4.075	0.801721	0.498	NA	0.024519	0.983857	27.2	0.6227
CYP2B6 (rs764288403)	19/41016652	ENST00000324071 (NA)	c.1301G>A	p.Arg434Gln	0.004	1	0.19486	NA	0.978638	0.54	0.947	0.159851	0.847492	26.4	0.5881
CYP27A1 (rs145722193)	2/218814105	ENST00000258415 (Yes)	c.1102G>T	p.Val368Leu	0.008	0.703	0.47742	2.88	0.013185	0.254	0.57	-0.3174	0.093965	20.1	0.1701
CYP27A1 (rs151117761)	2/218814998	ENST00000258415 (Yes)	c.1564G>A	p.Val522Met	0.003	0.998	0.44796	2.91	0.119885	0.506	NA	-0.26365	0.29709	24.5	0.2645

RED	Deleterious
GREEN	Tolerable
NA	Not available (Tool could not give prediction)

Table S3: Range and Predictive threshold of tools for testing variant deleteriousness.

Tool	Range	Predictive threshold
ClinPred	0-1	>0.5
MetaRNN		>0.5
BayesDel_addAF	-1.11707-0.750927	>0.0692655
REVEL	0-1	>0.5
CADD	0-50+	>20
AlphaMissense	0-1	Pathogenic (>=0.564-1), Ambiguous (0.34-0.56), Benign (<0.34)
MutPred	0-1	>0.5
PolyPhen2	0-1	>0.5
MutationAssessor	-5.17-6.49	High (>3.5), Medium (1.935-3.5). We used >=1.935 (to cater for both medium and high
MutationTaster_rankscore		>0.31733
SIFT	0-1	<0.05

Table S4: Minor allele frequency of variants

GENE	Genomic coordinate	Genotype of proband	HGVSc	HGVSp	MAF				
					AFR	ACB	ASW	YRI	Current Cohort
CYP1A2	15:74749955 (rs45565238)	G/A	NM_000761.5:c.217G>A	p.Gly73Arg	0.001	0.005	N/A	N/A	0.0103
	15:74750007 (**Novel**)	G/C	NM_000761.5:c.269G>C	p.Arg90Pro	N/A	N/A	N/A	N/A	0.0051
CYP2C18	10:94724372 (rs59636573)	G/T	NM_000772.3:c.988G>T	p.Val330Leu	0.015	0.026	0.008	0.014	0.0103
	10:94720472 (rs60181876)	C/T	NM_000772.3:c.896C>T	p.Thr299Ile	0.010	0.005	0.008	0.005	0.0103
CYP27A1	2:218814105 (rs145722193)	G/T	NM_000784.4:c.1102G>T	p.Val368Leu	0.006	N/A	0.008	N/A	0.0051
	2:218814998 (rs151117761)	G/A	NM_000784.4:c.1564G>A	p.Val522Met	0.001	N/A	N/A	N/A	0.0103
CYP2B6	19:41016652 (rs764288403)	G/A	NM_000767.5:c.1301G>A	p.Arg434Gln	2.987×10^{-5}	N/A	N/A	N/A	0.0051
	19:41004122 (rs372295360)	G/A	NM_000767.5:c.293G>A	p.Arg98Gln	0.00	N/A	N/A	N/A	0.0051
SLC6A2	16:55691982 (rs45564432)	C/G	NM_001043.3:c.848C>G	p.Thr283Arg	0.026	0.047	0.016	0.028	0.0103
ABCC3	17:50683692 (rs11568591)	G/A	NM_003786.4:c.3890G>A	p.Arg1297His	0.005	0.016	0.016	N/A	0.0154
	17:50677861 (rs34620384)	C/T	NM_003786.4:c.3496C>T	p.Arg1166Cys	0.005	0.010	N/A	N/A	0.0103

MAF = Minor allele frequency; AFR = Africa; ACB = African Caribbean in Barbados; ASW = African Ancestry in Southwest US; YRI = Yoruba in Ibadan.

Table S5: Syndromes, metabolic pathways and common substrates associated with genes with pathogenic variants.

**Variants of Gene**	**Name of syndrome (Inherited pattern)**	**Metabolic pathways**	**Drugs / Substrates**
CYP1A2<br>1. rs45565238<br>2. **Novel**	1. Porphyria Cutanea Tarda<br>2. Headache<br>3. Hepatocellular Adenoma<br>(Autosomal Dominant)	1. Imipramine/Desipramine Pathway<br>2. Biosynthesis of specialized proresolving mediators<br>3. Fatty acid metabolism	1. Caffeine<br>2.Ketoconazole<br>3. Carbamazepine<br>4. Fluvoxamine<br>5. Omeprazole
CYP2C18<br>1. rs59636573<br>2. rs60181876	1. Danubian Endemic Familial Nephropathy<br>2. Haemoglobin D Disease<br>3. Coumarin Resistance<br>(Autosomal dominant)	1. Oxidation by cytochrome P450<br>2. Statin Pathway - Generalized, P<br>3. Imipramine/Desipramine Pathway	1. Tolbutamide<br>2. Diclofenac<br>3. Omeprazole<br>4. Clobazam<br>5. Clotiazepam
CYP27A1<br>1. rs145722193<br>2. rs151117761	1. Xanthomatosis<br>2. Lipid Storage Disease<br>3. Rickets<br>4. Cholestasis<br>(Autosomal recessive)	1. Synthesis of bile acids and bile salts<br>2. Oxidation by cytochrome P450<br>3. Metapathway biotransformation Phase I and II<br>4. Diseases of glycosylation	1.Chenodeoxycholic acid<br>2. Cholecalciferol<br>3. Doxercalciferol<br>4. Ergocalciferol<br>5. Cholesterol
CYP2B6<br>1. rs764288403<br>2. rs372295360	1. Neonatal Abstinence Syndrome<br>2. Acute Frontal Sinusitis<br>3. Vitreous Abscess<br>(X linked dominant)	1. Tamoxifen and Artemisinin pathways<br>2. Methadone Pathway (Tramadol pharmacokinetics)<br>3. Nevirapine Pathway	1. Efavirenz<br>2. Nevirapine<br>3. Artemether<br>4. Acetaminophen<br>5. Chloramphenicol
SLC6A2<br>1. rs45564432	1. Orthostatic Intolerance Anxiety Disorder<br>2. Major Depressive Disorder<br>3. Attention Deficit-Hyperactivity Disorder<br>(Autosomal dominant)	1. Transport of inorganic cations/anions and amino acids/oligopeptides<br>2. Disorders of transmembrane transporters<br>3. Nuclear receptors meta-pathway<br>4. Venlafaxine Pathway	1. Atomoxetine<br>2. Milnacipran<br>3. Methylphenidate<br>4. Venlafaxine<br>5. Dextroamphetamine
ABCC3<br>1. rs11568591<br>2. rs34620384	1. Dubin-Johnson Syndrome<br>2. Cholestasis<br>3. Extrahepatic Cholestasis<br>4. Colon Adenocarcinoma<br>(Autosomal recessive)	1. Synthesis of bile acids and bile salts<br>2. Aspirin Metabolism<br>3. Codeine and Morphine Pathway<br>3. Nuclear events mediated by NFE2L2<br>4. Acetaminophen Pathway	1. Acetaminophen<br>2. Dexamethasone<br>3. Methotrexate<br>4. Indomethacin<br>5. Etoposide

Table S6: Other Variants observed in the 50 genes of interest.

Genomic coordinate	Gene Name	Genotype	HGVSc	HGVSp	Variant in father? (Genotype)	Variant in mother? (Genotype)	Number of tools that predicted pathogenicity
10:94762760 (rs17882687)	***CYP2C19***	A/C	NM_000769.4:c.55A>C	NP_000760.1:p.Ile19Leu	Yes (A/C)	None	1
10:94849995 (rs17879685)	***CYP2C19***	C/T	NM_000769.4:c.1228C>T	NP_000760.1:p.Arg410Cys	None	Yes (C/T)	0
17:50684036 (rs11568588)	***ABCC3***	C/T	NM_003786.4:c.4042C>T	NP_003777.2:p.Arg1348Cys	Yes (C/T)	None	4
2:218812299 (rs2229381)	***CYP27A1***	C/T	NM_000784.4:c.524C>T	NP_000775.1:p.Thr175Met	None	Yes (C/T)	0
16:16122020 (rs28706727)	***ABCC1***	G/A	NM_004996.4:c.3436G>A	NP_004987.2:p.Val1146Ile	None	Yes (G/A)	3
10:94981224 (rs28371685)	***CYP2C9***	C/T	NM_000771.4:c.1003C>T	NP_000762.2:p.Arg335Trp	None	Yes (C/T)	1
10:94852736 (rs146991374)	***CYP2C19***	A/T	NM_000769.4:c.1295A>T	NP_000760.1:p.Lys432Ile	Yes (A/T)	None	0
10:133538852 (rs28969387)	***CYP2E1***	A/T	NM_000773.4:c.1370A>T	NP_000764.1:p.His457Leu	None	Yes (A/T)	2

**Genomic coordinate**	**Gene Name**	**Genotype**	**HGVSc**	**HGVSp**	**Variant in father? (Genotype)**	**Variant in mother? (Genotype)**	**Number of tools that predicted pathogenicity**
8:18400612	***NAT2***	G/T	NM_000015.3:c.609G>T	NP_000006.2:p.Glu203Asp	None	Yes (G/T)	2
2:218812608 (rs149897566)	***CYP27A1***	G/A	NM_000784.4:c.703G>A	NP_000775.1:p.Glu235Lys	None	Yes (G/A)	1
2:218812281 (rs59443548)	***CYP27A1***	C/T	NM_000784.4:c.506C>T	NP_000775.1:p.Ala169Val	None	Yes(C/T)	1
10:99800412 (rs7080681)	***ABCC2***	G/A	NM_000392.5:c.1058G>A	NP_000383.2:p.Arg353His	None	Yes G/A	3
8:18400841 (rs56393504)	***NAT2***	G/A	NM_000015.3:c.838G>A	NP_000006.2:p.Val280Met	Yes (G/A)	None	2
10:94775106 (rs145328984)	***CYP2C19***	C/T	NM_000769.4:c.217C>T	NP_000760.1:p.Arg73Cys	None	Yes (C/T)	1
17:50661057 (rs45617731)	***ABCC3***	G/T	NM_003786.4:c.941G>T	NP_003777.2:p.Ser314Ile	Yes (G/T)	None	3
8:18400841 (rs56393504)	***NAT2***	G/A	NM_000015.3:c.838G>A	NP_000006.2:p.Val280Met	None	Yes (G/A)	3

**Genomic coordinate**	**Gene Name**	**Genotype**	**HGVSc**	**HGVSp**	**Variant in father? (Genotype)**	**Variant in mother? (Genotype)**	**Number of tools that predicted pathogenicity**
10:94781858 (rs6413438)	***CYP2C18***	C/T	NM_000769.4:c.680C>T	NP_000760.1:p.Pro227Leu	None	Yes (C/T)	0
1:46799184 (rs55672106)	***CYP4B1***	C/T	NM_001099772.2:c.103C>T	NP_001093242.1:p.Arg35Trp	None	Yes (C/T)	1
7:5299105 (rs150889269)	***SLC29A4***	C/T	NM_153247.4:c.1000C>T	NP_694979.2:p.Arg334Cys	None	Yes (C/T)	2
8:18400641 (rs138707146)	***NAT2***	C/T	NM_000015.3:c.638C>T	NP_000006.2:p.Pro213Leu	Yes (C/T)	None	0
19:40991369 (rs8192709)	***CYP2B6***	C/T	NM_000767.5:c.64C>T	NP_000758.1:p.Arg22Cys	None	Yes (C/T)	3
10:94849995 (rs17879685)	***CYP2C19***	C/T	NM_000769.4:c.1228C>T	NP_000760.1:p.Arg410Cys	Yes (C/T)	None	4
19:40991381	***CYP2B6***	A/T	NM_000767.5:c.76A>T	NP_000758.1:p.Thr26Ser	Yes (A/T)	None	0
8:18400841 (rs56393504)	***NAT2***	G/A	NM_000015.3:c.838G>A	NP_000006.2:p.Val280Met	YES (G/A)	None	3

**Genomic coordinate**	**Gene Name**	**Genotype**	**HGVSc**	**HGVSp**	**Variant in father? (Genotype)**	**Variant in mother? Genotype)**	**Number of tools that predicted pathogenicity**
2:218812281 (rs59443548)	***CYP27A1***	C/T	NM_000784.4:c.506C>T	NP_000775.1:p.Ala169Val	None	Yes (C/T)	1
7:5287923	***SLC29A4***	C/T	NM_153247.4:c.107C>T	NP_694979.2:p.Ala36Val	None	Yes (C/T)	0
10:94849995 (rs17879685)	***CYP2C19***	C/T	NM_000769.4:c.1228C>T	NP_000760.1:p.Arg410Cys	Yes (C/T)	None	2
7:5299105 (rs150889269)	***SLC29A4***	C/T	NM_153247.4:c.1000C>T	NP_694979.2:p.Arg334Cys	None	Yes (C/T)	4
16:16114826 (rs13337489)	***ABCC1***	G/C	NM_004996.4:c.3140G>C	NP_004987.2:p.Cys1047Ser	None	Yes (G/C)	1
15:74750299	***CYP1A2***	C/G	NM_000761.5:c.561C>G	NP_000752.2:p.Asp187Glu	Yes(C/G)	None	4
17:50658456	***ABCC3***	G/T	NM_003786.4:c.634G>T	NP_003777.2:p.Ala212Ser	Yes (G/T)	None	0
10:94762760 (rs17882687)	***CYP2C19***	A/C	NM_000769.4:c.55A>C	NP_000760.1:p.Ile19Leu	Yes (A/C)	Yes (A/C)	3

**Genomic coordinate**	**Gene Name**	**Genotype**	**HGVSc**	**HGVSp**	**Variant in father? (Genotype)**	**Variant in mother? (Genotype)**	**Number of tools that predicted pathogenicity**
8:18400475 (rs139351995)	***NAT2***	A/C	NM_000015.3:c.472A>C	NP_000006.2:p.Ile158Leu	None	Yes (A/C)	1
16:16114826 (rs13337489)	***ABCC1***	G/C	NM_004996.4:c.3140G>C	NP_004987.2:p.Cys1047Ser	None	Yes (G/C)	1
8:18400406 (rs12720065)	***NAT2***	C/G	NM_000015.3:c.403C>G	NP_000006.2:p.Leu135Val	Yes (C/G)	None	3
10:94762760 (rs17882687)	***CYP2C19***	A/C	NM_000769.4:c.55A>C	NP_000760.1:p.Ile19Leu	Yes (A/C)	None	2
10:94775197	***CYP2C19***	C/A	NM_000769.4:c.308C>A	NP_000760.1:p.Ala103Asp	Yes (C/A)	None	2
19:40991369 (rs8192709)	***CYP2B6***	C/T	NM_000767.5:c.64C>T	NP_000758.1:p.Arg22Cys	Yes (C/T)	None	0
10:99800412 (rs7080681)	***ABCC2***	G/A	NM_000392.5:c.1058G>A	NP_000383.2:p.Arg353His	YES (G/A)	None	2
16:16114826 (rs13337489)	***ABCC1***	G/C	NM_004996.4:c.3140G>C	NP_004987.2:p.Cys1047Ser	None	Yes (G/C)	3
2:218812281 (rs59443548)	***CYP27A1***	C/T	NM_000784.4:c.506C>T	NP_000775.1:p.Ala169Val	None	Yes (C/T)	0

**Genomic coordinate**	**Gene Name**	**Genotype**	**HGVSc**	**HGVSp**	**Variant in father? (Genotype)**	**Variant in mother? (Genotype)**	**Number of tools that predicted pathogenicity**
17:50656775 (rs35777968)	***ABCC3***	G/A	NM_003786.4:c.296G>A	NP_003777.2:p.Arg99Gln	None	Yes (G/A)	0
10:94762760 rs17882687	***CYP2C19***	A/C	NM_000769.4:c.55A>C	NP_000760.1:p.Ile19Leu	Yes (A/C)	None	4
19:15551328 (rs150443867)	***CYP4F22***	T/G	NM_173483.4:c.1453T>G	NP_775754.2:p.Leu485Val	None	Yes (T/G)	0
17:50656775 (rs35777968)	***ABCC3***	G/A	NM_003786.4:c.296G>A	NP_003777.2:p.Arg99Gln	None	Yes(G/A)	0
10:94781858 (rs6413438)	***CYP2C19***	C/T	NM_000769.4:c.680C>T	NP_000760.1:p.Pro227Leu	None	Yes (C/T)	1

Table S7: Binding affinity of ligands to *CYP1A2* wildtype and variants.

**Ligand**		**Binding Affinity**		
**Name**	**3D Structure**	**Wildtype**	**Gly73Arg**	**Arg90Pro**
Caffeine	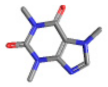	-7.2	-6.9	-6.0
Ketoconazole	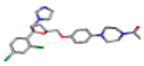	-8.1	-8.0	-8.0
Carbamazepine	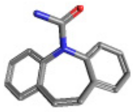	-8.4	-8.4	-8.4
Fluvoxamine	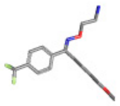	-7.7	-6.2	-6.0
Omeprazole	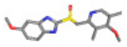	-8.5	-9.0	-9.0

Table S8: Binding affinity of *CYP2C18* wildtype and variants to ligands.

**Ligand**		**Binding Affinity**		
**Name**	**3D Structure**	**Wildtype**	**Val330Leu**	**Thr299Ile**
Tolbutamide	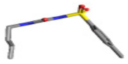	-6.1	-5.9	-6.9
Diclofenac	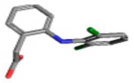	-7.6	-7.5	-7.6
Omeprazole	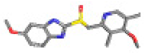	-7.7	-6.9	-7.7
Clobazam	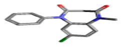	-6.7	-6.7	-6.6
Clotiazepam	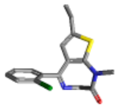	-6.7	-6.3	-6.3

Table S9: Binding affinity of *CYP27A1* wildtype and variants to ligands.

Ligand		Binding Affinity		
**Name**	**3D Structure**	**Wildtype**	**Val368Leu**	**Val522Met**
Chenodeoxycholic acid	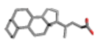	-8.8	-7.7	-9.1
Cholecalciferol	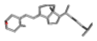	-9.1	-8.3	-9.4
Doxercalciferol	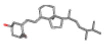	-9.7	-9.8	-9.8
Ergocalciferol	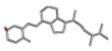	-9.6	-9.9	-9.6
Cholesterol	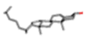	-9	-9.6	-9.5

Table S10: Binding affinity of *CYP2B6* wildtype and variants to ligands.

Ligand		Binding Affinity		
**Name**	**3D Structure**	**Wildtype**	**Arg434Gln**	**Arg98Gln**
Efavirenz	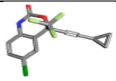	-8.3	-7.3	-7.2
Nevirapine	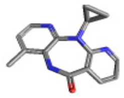	-6.6	-6.5	-8.4
Artemether	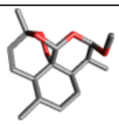	-6.9	-6.9	-7.0
Acetaminophen	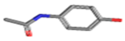	-5.3	-5.5	-5.9
Chloroamphinecol	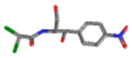	-5.8	-5.8	-7.1

Table S11: Binding affinity of *SLC6A2* wildtype and variant to ligands.

Ligand		Binding Affinity	
**Name**	**3D Structure**	**Wildtype**	**Thr283Arg**
Atomoxetine	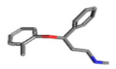	-6.9	-6.9
Milnacipran	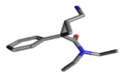	-6.2	-6.2
Methylphenidate	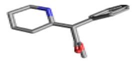	-6.3	-6.2
Venlafaxine	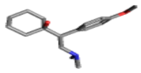	-6.3	-6.4
Dextroampetamine	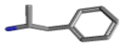	-6.7	-6.7

Table S12: Binding affinity of *ABCC3* wildtype and variants to ligands.

Ligand		Binding Affinity		
**Name**	**3D Structure**	**Wildtype**	**Arg1297His**	**Arg1166Cys**
Acetaminophen	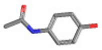	-5.6	-5.7	-5.6
Dexamethasone	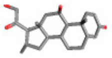	-8.0	-8.0	-8.6
Methotrexate	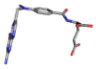	-8.9	-9.3	-8.4
Indomethacin	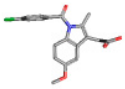	-7.4	-7.1	-7.1
Etoposide	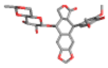	-8.9	-9.1	-8.6
